# Pay for Performance: A Reflection on How a Global Perspective Could Enhance Policy and Research

**DOI:** 10.34172/ijhpm.2020.23

**Published:** 2020-02-24

**Authors:** Laura Anselmi, Josephine Borghi, Garrett Wallace Brown, Eleonora Fichera, Kara Hanson, Artwell Kadungure, Roxanne Kovacs, Søren Rud Kristensen, Neha S. Singh, Matt Sutton

**Affiliations:** ^1^Health, Organisation, Policy and Economics (HOPE), Centre for Primary Care and Health Service Research, Faculty of Biology, Medicine and Health, University of Manchester, Manchester, UK.; ^2^Department of Global Health and Development, Faculty of Public Health and Policy, London School of Hygiene and Tropical Medicine, London, UK.; ^3^School of Politics and International Studies (POLIS), University of Leeds, Leeds, UK.; ^4^Department of Economics, University of Bath, Bath, UK.; ^5^Training and Research Support Centre (TARSC), Harare, Zimbabwe.; ^6^Centre for Health Policy, Institute of Global Health Innovation, Imperial College London, London, UK.

**Keywords:** Health Financing, Pay-For-Performance, Comparative Research

## Abstract

Pay-for-performance (P4P) is the provision of financial incentives to healthcare providers based on pre-specified performance targets. P4P has been used as a policy tool to improve healthcare provision globally. However, researchers tend to cluster into those working on high or low- and middle-income countries (LMICs), with still limited knowledge exchange, potentially constraining opportunities for learning from across income settings. We reflect here on some commonalities and differences in the design of P4P schemes, research questions, methods and data across income settings. We highlight how a global perspective on knowledge synthesis could lead to innovations and further knowledge advancement.

Pay-for-performance (P4P), or the provision of financial incentives to healthcare providers based on pre-specified performance targets, first emerged as a strategy to improve quality of care in the United States, Europe, and other high-income countries (HICs), and was subsequently adopted in low- and middle-income countries (LMICs) with the further aim of increasing service coverage.^1,2^ While this approach to provider payment is now a global phenomenon, the community of health economists and health service researchers working on P4P tends to divide into those concentrating on HICs, and those concentrating on LMICs. Literature reviews on the topic also tend to focus on one of these settings.^1-8^ To date, little effort has been made to look at the global evidence on P4P, including the research questions, methods and types of data used to study P4P, the research findings, and how and why these vary across income settings. Most importantly, there has been little reflection on whether there is variation in the use of P4P as a policy intervention, and on its effectiveness and mechanisms of effect in HICs compared to LMICs. This lack of connection between the two groups of researchers and their research agendas may present a missed opportunity to improve research methods and gain a common understanding of where, why and under which circumstances P4P may work.

From our contributions to recent academic conferences and policy debates, and from our collective knowledge of the literature, we have identified a number of issues related to P4P currently studied in different settings and for which a global perspective could bring new insights. These include the specifics of incentive design, mechanisms of effect, spillover effects, context moderators, and sustainability of P4P over time. In addition, there are important differences in the way that research is undertaken, including research design, methods and data used. And finally, there are key areas where deliberately comparative research that sets out to study these topics across high and low-income settings, could potentially lead to innovations and knowledge advancement. We review differences in research questions, research design, methods and data used to highlight where researchers could reflect on adopting research elements used from colleagues working in other settings to innovate, generate new knowledge and create a common ground for cross-learning.

## The Specifics of Incentive Design

The majority of research so far has focused on understanding the effect of P4P on healthcare delivery and health outcomes.^1-8^ What is lacking in both settings, is a better understanding of the design features that contribute to, or undermine, the effectiveness of P4P with respect to their objectives.^9,10^ Indeed, few recent studies investigate and highlight the relevance of incentive design elements that enabled P4P to achieve intended outcomes, and its potential spillovers to non-incentivised service areas.^11-13^ In both settings, there is a need for a deeper and broader understanding of the interplay between design and context that determines programme effects and their sustainability over time. A clear description of the design in relation to pre-defined typologies would be a first research element that could facilitate cross settings analysis,^9,10^ including understanding how the context shapes policy objectives and design and how these broadly differ across settings. Differences in objectives and design will inevitably shape the way research is done, especially considering that in LMIC P4P may have been implemented with the view to reform the overall healthcare system.^10,14^

## Mechanisms and Spillovers

Understanding the mechanisms through which P4P operates is also important to answer questions on the appropriate use of P4P schemes.^15-17^ Key questions include when and for which policy objectives P4P schemes can be applied, as well as which design features can be chosen by policy-makers to obtain long-term, sustainable changes. For example, schemes may be associated with improvements in care processes and care use, but not associated with improved health outcomes.^5,18,19^ They can also be associated with changes in incentivised processes and outcomes, including for example diversion away from incentivized tasks or improvement of performance on complementary tasks.^20^ There is also a risk of unintended performance reduction when incentives are reduced or removed.^21^ Understanding why and when this happens, and if it is a consequence of scheme design or implementation,^22,23^ can help to determine whether incentives can be used temporarily as a way to prompt long-term behavioural changes, or whether a sustained increase in funding is needed.^24^ And again the answers may be different in HIC or LMIC, where health services are more severely underfunded.

## Context

Context strongly influences not only the implementation process, but also the way schemes are designed, in terms of the political actors involved in its conception, the overall aims and the focus of target setting. In England and Scotland, for instance, P4P was introduced by the National Health Service, with the objective of improving the quality of either primary or secondary care provided to patients, or patients with specific conditions.^12^ In contrast, in LMICs, most schemes have been driven and funded by international aid and reflect the priorities of donors, as well as those of the recipient government, often with a shift from input to output based financing.^9^ Although adapted to local circumstances,^25^ funding and targets are concentrated on selected healthcare indicators with high priority in the international agenda, with the primary objective of increasing access to care and making healthcare purchasing more strategic.^26,27^ The setting also affects performance. Even within the same setting, providers exposed to different contextual factors appear to achieve different levels of performance, at least in the short term.^28,29^

In low- and middle-income settings, where absolute levels of health spending are much lower and international aid may represent a high share of the total spending, P4P funding can be crucial to the functioning of the healthcare system. The bonus payments received are often either re-invested in supporting the current expenditure of the healthcare provider, and/or paid directly to health workers to augment their remuneration,^30,31^ or serve as an effective salary payment. In high-income settings health workers rarely benefit directly from any performance related payments. This difference could influence how providers respond to incentives and to additional payments, or their removal. For example in low-income settings, the removal of bonus payments may lead to a drop in service provision (ie, increased absenteeism),^32^ whereas in better funded systems this may only affect the sustainability of process improvements.^22^

## Research Focus and Design

Differences in the nature of health systems and in the reasons for introducing P4P between HIC and LMIC have had some influence over the focus and design of the research. For example, in HIC questions about the sustainability of the effects of P4P over the longer term have been explored more extensively, possibly due to the longer history of implementing P4P and more consolidated routine information systems.^33-35^ In contrast, in LMICs where experience with P4P is more recent and information systems are weaker, effects have typically been assessed at one point in time within impact evaluation studies, with only some consideration of the longer term financial sustainability of these schemes, which are initially dependent on donor funding.^8,9,36,37^

Furthermore, P4P schemes implemented in low- and middle-income settings are often set up as part of a bundle of interventions to reform the health system, through strengthening health information systems, enhancing provider autonomy, and promoting greater financial decentralisation.^9,38^ As a result some of the LMIC research has sought to examine the interactions between P4P and the broader health system building blocks involved in the achievement of these targets.^15-17,39^ However, in HIC P4P does not typically require broader reforms to the health system, and hence system level effects of P4P are less commonly studied. In England, for example, the interaction between P4P schemes and the way public funding is managed (ie, how budgets are allocated to different sectors and public institutions, and then executed to deliver services) has rarely been examined. For example, rigid centralised financial management and procurement can act as a barrier to budget execution and service delivery.^27^ In high-income settings where P4P is implemented in the public sector, the centralised public finance management is not considered by P4P researchers, perhaps because it does not represent a constraint. Both LMIC and HIC studies would benefit from considering the wider financing architecture within which P4P is embedded and the effect this has on programme success.^38^

In both low- and high-income settings, existing research has not systematically accounted for the interplay between P4P and other policies, and between P4P payments and the allocation of other sources of funding for providers, even if these have been shown to exist.^27^ In particular, the opportunity costs of financing these schemes is often not considered. The assessment of the cost-effectiveness of P4P, as well as other health policies and health system interventions, is still in its infancy in both settings, with potentially controversial assumptions and methods needing refinement, but remains crucial.^8,36,40-42^ The assessment of whether programme benefits are concentrated in certain population groups has been explored more extensively by researchers in HIC^43-45^ and there are growing numbers of studies examining this issue in LMIC,^28,46^ but more evidence could be helpful to address potential equity concerns.^47^

## Research Methods and Data

The methods and data used to support P4P research has also differed between high- and low-income settings. In low- and middle-income settings research often relies on the use of mixed methods and includes careful process evaluations.^24,27^ This is perhaps a response to the need to understand not only the context, and the interaction between P4P schemes and the healthcare and public services system as a whole, but also how to set up data collection. In high-income settings where information systems are more structured and consolidated, single methods analyses are more common.

In low- and middle-income settings routine healthcare data is often weak, with missing data and incomplete time series. Therefore, impact evaluations are more likely to be based on randomised control or quasi-experimental studies, with significant primary data collection. This facilitates analysis of the impact of P4P on a wide range of effects as hypothesized in a programme theory of change, including an assessment of potential mediators of programme effect. In contrast, in high-income settings, routine data systems support rigorous observational studies measuring impacts on a more limited range of outcomes over a longer period of time.^48^ Research informed by routine data may overlook effects on important outcomes that are not available in administrative systems.

## The Value of Taking a Global Perspective

Every healthcare system is different. These differences will inevitably shape both the goals, design and implementation of P4P schemes as well as the research that is undertaken. However, there is value in looking across settings both at policy experience, research methods and findings to improve our understanding of which interventions, such as P4P, may strengthen health systems, and of how to inform best practice. Looking across settings involves adopting a pluralistic perspective, which allows for greater variation in processes of evidence synthesis, verification and communication. Doing so will help to support further critical scrutiny on research ethics, quality, interpretation and uptake, and ultimately higher ethical accountability of policy-makers.^49^

A growing number of papers synthesizing evidence across similar settings is a valuable step forward in this regard.^1-8^ We argue that, beyond the value of learning across disciplines and programmes,^9^ we can gain even more by breaking out geographic silos and looking at lessons learnt from across low- and middle- and high-income settings. Given similarities and differences that we highlighted, a few examples emerge. Research on the design of programmes and how design and context affect impact is of global relevance and can only be undertaken by looking across settings. More analysis of spillovers and heterogeneous effects may be done in LMIC using methods and frameworks applied in HIC. On the other hand, there is room to extend the analysis of mechanisms and health system effects, which is already growing in LMIC. As routine data systems improve in LMIC they can be used to perform the type of analyses already performed in HIC, and similarly HIC researchers may consider ideas for primary research in LMIC settings.

Concrete steps may be undertaken at the structural level by international organizations, such as the World Health Organization (WHO) working on the political economy of health financing reforms.^50^ The WHO could create forums for discussion and opportunities for comparative research, as well as promote the use of overarching frameworks that may facilitate comparisons and information exchange. Academic journals could also systematically encourage efforts to bridge the gaps across research communities. For example they could encourage reviews to consider global rather than a geographic subset of the literature and examine the relevance of context to the findings and require for studies reporting new evidence a discussion of how research design, methods, data and findings may differ from those in other settings. Such initiatives, may also further contribute to promote evidence based policy-making, increase research quality and prevent conflict of interests.

Despite the obvious contextual differences of doing health policy research in different income settings, the focus on common themes and research insights emerging from these different settings will undoubtedly lead to a fuller understanding of the topic we share a research interest in and improve our research practice. Concrete steps may also be taken by individual researchers by looking from HIC to LMIC and vice versa, in addition to comparing findings with those from similar economic setting. We invite more researchers to join us in this endeavour.

## Acknowledgements

We acknowledge funding for the PEMBA (Performance-based financing mechanisms for health system strengthening in Africa) project, jointly funded by the Medical Research Council, Economic and Social Research Council, Department for International Development and the Wellcome Trust through the Health System Research Initiative (Grant ref: MR/P014429/1).

We acknowledge the presenters and participants in the one-day workshop at the Fifth Global Symposium on Health Systems Research: Advancing Health Systems for All in the Sustainable Development Goals Era in Liverpool in October 2018, organised by the authors (who are collaborators in the PEMBA project) to reflect on the themes of incentive design, spillover effects, context moderation, and sustainability of P4P. We are particularly grateful to presenters, discussants and chairs: Martin Chalkley [University of York, UK], Rachel Foskett-Tharby [NHS England, UK], Kunle Alonge [Johns Hopkins University, USA], Wiktoria Tafesse [University of York, UK], Ivdity Chikovani [Curatio International Foundation, Georgia], Stefanie Tan [LSHTM, UK], Sophie Witter [Queen Margaret University, UK], Stephen Peckham [University of Kent, UK], Philip Britteon [University of Manchester, UK], Jeffery C. Tanner [World Bank], Søren Rud Kristensen [Imperial College London, UK], Valéry Ridde [Universités Paris Sorbonne Cités and IRD (French Institute For Research on sustainable Development), France], James Gaughan [University of York, UK], Peter Binyaruka [Ifakara Health Institute, Tanzania], Maria Bertone [Queen Margaret University, UK], Manassé Nimpagaritse [Institut National de Santé Publique, Burundi], Ruth McDonald [University of Manchester, UK], Fabiana Saddi [Federal University of Goiás, Brazil and CHSS/University of Kent, UK], Bruce Guthrie [University of Dundee, UK], Anna Wilding [University of Manchester, UK], Oriane Bodson [University of Liege, Belgium], Christos Grigoroglou [University of Manchester, UK], Tim Doran [University of York, UK].

## Ethical issues

Not Applicable.

## Competing interests

Authors declare that they have no competing interests.

## Authors’ contributions

LA drafted the manuscript. LA, JB, GWB, EF, KH, AK, RK, SRK, NSS, and MS critically revised the manuscript for important intellectual content. All authors approved the final version of the manuscript.

## Authors’ affiliations

^1^Health, Organisation, Policy and Economics (HOPE), Centre for Primary Care and Health Service Research, Faculty of Biology, Medicine and Health, University of Manchester, Manchester, UK. ^2^Department of Global Health and Development, Faculty of Public Health and Policy, London School of Hygiene and Tropical Medicine, London, UK. ^3^School of Politics and International Studies (POLIS), University of Leeds, Leeds, UK. ^4^Department of Economics, University of Bath, Bath, UK. ^5^Training and Research Support Centre (TARSC), Harare, Zimbabwe. ^6^Centre for Health Policy, Institute of Global Health Innovation, Imperial College London, London, UK.
